# Developments in optics and performance at BL13-XALOC, the macromolecular crystallography beamline at the Alba Synchrotron

**DOI:** 10.1107/S160057751400825X

**Published:** 2014-05-20

**Authors:** Jordi Juanhuix, Fernando Gil-Ortiz, Guifré Cuní, Carles Colldelram, Josep Nicolás, Julio Lidón, Eva Boter, Claude Ruget, Salvador Ferrer, Jordi Benach

**Affiliations:** a ALBA Synchrotron, BP 1413, km 3.3, Cerdanyola del Vallès, Spain; b MAX IV Laboratory, Ole Römers väg 1, 223 63 Lund, Sweden; c Fusion for Energy, Josep Pla 2, 08019 Barcelona, Spain

**Keywords:** macromolecular crystallography, beamline, Alba, mirror slope errors

## Abstract

BL13-XALOC is currently the only macromolecular crystallography beamline at the 3 GeV ALBA synchrotron near Barcelona, Spain. The optics design is based on an in-vacuum undulator, a Si(111) channel-cut crystal monochromator and a pair of KB mirrors. It allows three main operation modes: a focused configuration, where both mirrors can focus the beam at the sample position to 52 µm × 5.5 µm FWHM (H × V); a defocused configuration that can match the size of the beam to the dimensions of the crystals or to focus the beam at the detector; and an unfocused configuration, where one or both mirrors are removed from the photon beam path. To achieve a uniform defocused beam, the slope errors of the mirrors were reduced down to 55 nrad RMS by employing a novel method that has been developed at the ALBA high-accuracy metrology laboratory. Thorough commissioning with X-ray beam and user operation has demonstrated an excellent energy and spatial stability of the beamline. The end-station includes a high-accuracy single-axis diffractometer, a removable mini-kappa stage, an automated sample-mounting robot and a photon-counting detector that allows shutterless operation. The positioning tables of the diffractometer and the detector are based on a novel and highly stable design. This equipment, together with the operation flexibility of the beamline, allows a large variety of types of crystals to be tackled, from medium-sized crystals with large unit-cell parameters to microcrystals. Several examples of data collections measured during beamline commissioning are described. The beamline started user operation on 18 July 2012.

## Abstract 2

The design, performance and first results of the new macromolecular crystallography beamline BL13-XALOC at the ALBA Synchrotron are described, including new developments in optics and control.

## 1. Introduction   

**Figure 1:**
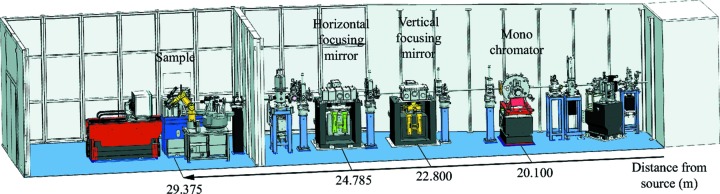
Overall layout of the BL13-XALOC beamline.

ALBA is a third-generation synchrotron light source built in Barcelona, Spain, with a 268 m storage ring circumference, 3 GeV electron energy and 4.6 nm rad measured emittance, and has been routinely operating since 2011 (Einfeld, 2011 ▶). The storage ring is at present operating at a current of 120 mA in decay uniform-filling mode, although it is foreseen to operate in top-up mode in 2014, and to increase the current to 250 mA gradually over the next two years. The portfolio of the seven phase-I beamlines includes beamline BL13, also called XALOC, that is dedicated to macromolecular crystallography (MX). Currently being the only MX beamline at ALBA, XALOC has been designed to deal not only with easily automatable X-ray diffraction experiments of well diffracting medium-sized crystals, but also with non-standard and more complex ones that include a variety of crystal sizes and unit-cell length dimensions, crystals with high mosaic spread, and/or poorly diffracting crystals. The beamline is also able to perform standard energy-dependent MX experiments (SAD/MAD) in the 5–22 keV energy range. The design guidelines of BL13-XALOC contrast with the trend observed lately in other synchrotrons, where MX beamlines target specific characteristics of the crystals (microcrystals, large unit cells), techniques (tunability, small or large wavelengths) or to the status of the MX projects (screening crystal diffraction). In order to have a reliable all-in-one beamline, two main design guidelines have been followed: stability and flexibility. As described later, these guidelines impose strict conditions on the optical system and the mechanics. On one hand, a high level of beam stability has been achieved by using relatively simple but effective designs for the optics and the end-station (Fig. 1 ▶), characterized by extensive vibrational and thermal finite element analysis (FEA), and systematic high-accuracy metrology on all critical components. On the other hand, we have remained flexible enough to easily change the beam size at the sample position without significantly affecting the beam path. The combination of the high levels of stability and flexibility allows us to operate effortlessly the beamline in two main modes, letting the user choose either to focus or defocus the beam at the sample position. When defocusing, the beam focus in either dimension can be adjusted from 1 m before the sample position (overfocusing) to infinite (unbending the mirror), and in particular at the detector position. A third operating mode, albeit more complex to set up, is also possible by removing the mirrors and thus *unfocusing* the beam, which involves varying the beam path by 53 mm in the vertical direction and/or 37 mm in the horizontal direction, a configuration that both the optics and the end-station are designed to cope with. This flexibility in the beam size without losing flux is relevant to better manage the radiation damage produced in the crystal. Although microbeams of <10 µm diminish the radiation damage per absorbed dose (Nave & Hill, 2005 ▶; Cowan & Nave, 2008 ▶; Sanishvili *et al.*, 2010 ▶), at the same time it has been shown that matching the dimensions of the beam to the size of the crystal improves the ratio between the diffracted intensities and the background scattering, which allows us to better estimate the radiation damage, provided that the beam illuminates uniformly the crystal (Bourenkov & Popov, 2006 ▶; Holton, 2009 ▶; Garman, 2010 ▶). The operational flexibility of XALOC allows us then to choose the strategy to minimize the radiation damage for each crystal, depending on its particular morphology or the visibility in the loop.

ALBA and the XALOC beamline have been constructed by CELLS (The Consortium for Construction, Equipment and Exploitation of the Synchrotron Light Laboratory), a consortium that receives its funding in equal parts from the Spanish government (*via* the Science Ministry) and the Catalan regional government or Generalitat de Catalunya (*via* the Department of Research and Universities). Beam time at XALOC is allocated to academic users worldwide *via* peer-reviewed proposals submitted through the ALBA web portal (http://useroffice.cells.es/). Beam time is also sold to industrial users.

## 2. Source and optics design   

The photon source of XALOC is a 2 m-long pure-permanent-magnet in-vacuum undulator (IVU21) manufactured by Bruker Advanced Supercon (formerly ACCEL) (Bergisch Gladbach, Germany) with a minimum gap of 5.7 mm and placed in a medium straight section of the storage ring (Doelling *et al.*, 2008 ▶; Campmany *et al.*, 2013 ▶) (Table 1 ▶). The photon source size and the horizontal divergence are limited by the electron beam emittance, whereas the vertical divergence is mainly limited by the electron energy spread. The parameters of IVU21 were chosen to have full tunability between 5 and 22 keV and to maximize the flux at the Se *K*-edge (12.658 keV), a common energy in MX experiments. The front-end includes a four-blade X-ray beam position monitor and the only moveable white-beam masks of the beamline. The first element of the beamline optics is a 400 µm-thick diamond window, which separates the front-end and the beamline vacuum sectors. The diamond window is placed at 17.2 m from the photon source, has an 8 mm-diameter aperture and absorbs most of the beam power below 5 keV.

The optical design, that has been kept identical to the one presented in the conceptual report of the beamline (Juanhuix & Ferrer, 2007 ▶), is constrained by the flexibility and stability guidelines. The use of an undulator eliminates the need of a collimating mirror before the monochromator, as its vertical beam divergence matches the rocking-curve width of the Si(111) reflection. Also, a toroidal mirror was not considered to refocus the beam because its horizontal focusing adjustment would have changed the incidence angle, modifying the beam path and, as a result, increased the complexity of the beamline. Moreover, a channel-cut Si(111) double-crystal monochromator (DCM) was chosen over a DCM with separate crystals because of the reduced complexity of the adjusting mechanics, which favours the beam stability. Finally, the use of compound refractive lenses and multilayer mirrors was discarded due to their dispersive focusing properties. Following these considerations, the main optical elements consist of a channel-cut monochromator followed by a pair of plane mirrors elliptically bent along the meridional direction, each focusing in either vertical and horizontal directions.

The optics of the beamline also includes a single-crystal 200 µm-thick diamond Laue monochromator, which is designed to deliver a 9.041 keV X-ray beam to a future side branch. At this side branch the beam can be focused to 750 µm × 550 µm FWHM (H × V) without any additional optics due to the focusing properties of the Laue diffraction (Sánchez del Río *et al.*, 1995 ▶). This monochromator is currently used as a white-beam filter since this side branch has not been built. More details can be found elsewhere (Juanhuix & Ferrer, 2007 ▶).

Several X-ray beam diagnostics have been implemented along the beam. The diagnostics in the optical hutch consist of two diamond fluorescence screens (FMB-Oxford, Oxford, UK) that are placed at the beginning (before the monochromator) and at the end of the optics system (after the KB mirrors) and four four-diode fluorescing-foil beam position monitors (BPMs) built in-house. The BPMs are designed to accommodate a hole grid to perform wavefront analysis using the Hartmann method (Mercère *et al.*, 2006 ▶) if required. The diagnostics in the experimental hutch include thin Si PIN-diodes, CVD diamond four-quadrant BPMs (Dectris, Baden, Switzerland), a Ce:YAG fluorescence screen, and a retractile PIN-diode at the sample position built in-house. All these diagnostics except the retractile diode are transmissive, with a transmission ranging between 80 and 95% at 12.4 keV for each element.

## 3. Monochromator   

**Figure 2:**
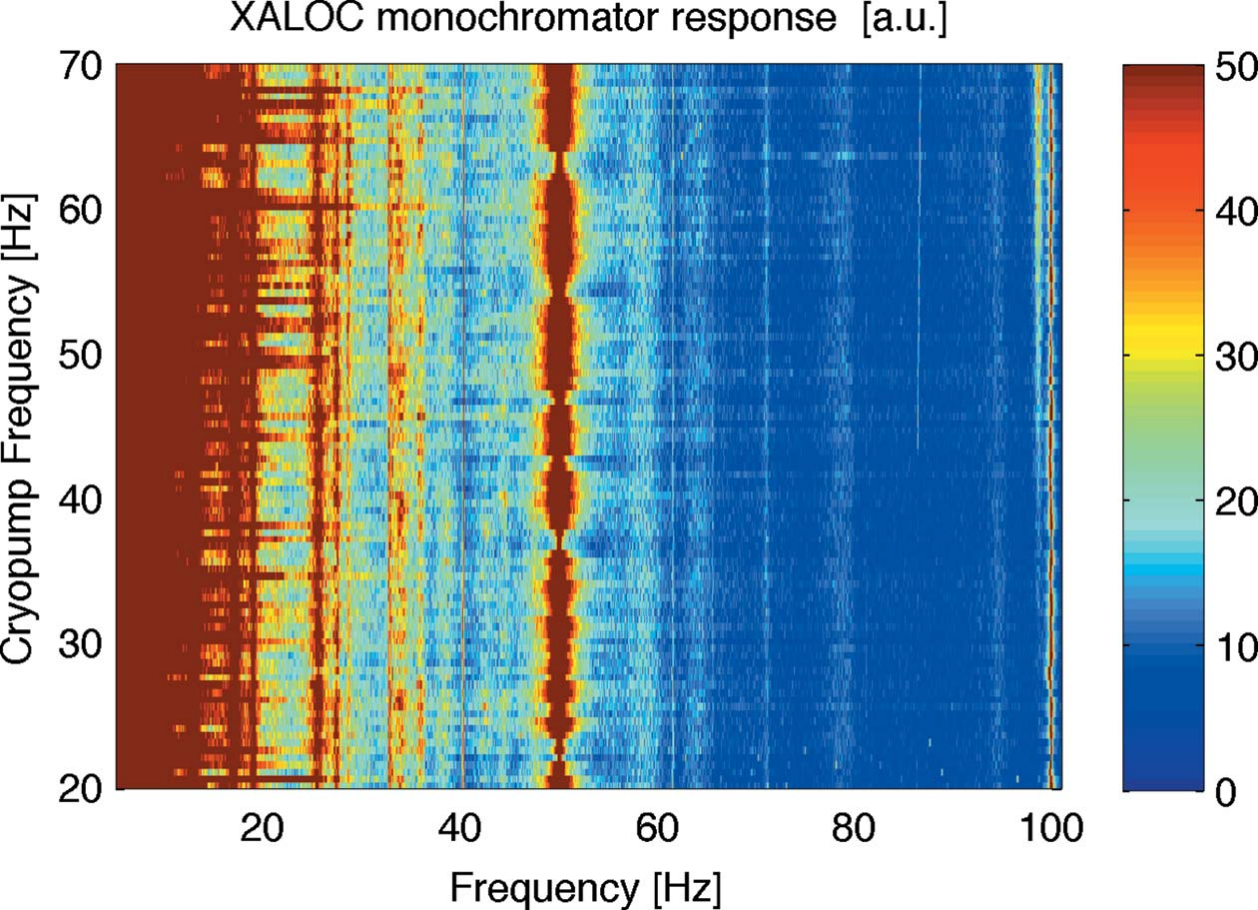
Vibrational behaviour of the monochromator as measured by laser interferometry, with the laser beam following the X-ray beam path, at a cryopump frequency that varied from 20 to 70 Hz (in 1 Hz steps). The excited frequencies (*e.g.* 19, 25, 50 Hz) are independent of the cryopump regime, and are due to resonances of the metrology set-up and the electronic noise.

The monochromator is a cryogenically cooled channel-cut DCM that uses the reflection (111) from Si and was manufactured by Cinel s.r.l. (Vigonza, Italy). Since stability is a main drive of the design, FEA was carried out to evaluate the effect of the heat load on the surface of the first monochromator crystal. The analysis was carried out assuming a power load of 155 W, which corresponds to a current of 250 mA in the storage ring and an aperture of the front-end slits that accepts totally the full central cone of the undulator. The FEA analysis shows that the meridional RMS slope error of the first crystal surface induced by heat load is reduced three-fold when the temperature of the liquid N_2_ (*T*_LN2_) is increased from 78 K to 90 K, which is the highest temperature at which the cryocooler was adjusted while preserving the vapour pressure well below the limit of operation (5 bar). Also, according to this FEA analysis, the temperatures at the first crystal surface are 97 K and 111 K, respectively. The reduction of the RMS slope error when increasing *T*_LN2_ is explained by the reduction of the absolute value of the silicon thermal expansion coefficient α, which is zero at ∼124 K. In view of these results, the cryocooling system should not be optimized to increase heat exchange, but rather to reduce vibrations. Several actions were taken in this direction aiming to bring the LN_2_ flux closer to a laminar regime. Firstly, the LN_2_ circuit in the heat exchanger that is in contact with the Si crystal was modified by computational fluid dynamics simulations in order to reduce the Reynolds number and to make the flow speed more uniform in the microchannels of the heat exchanger. Secondly, the average speed in the microchannels was reduced to 0.4 m s^−1^ or lower. Thirdly, the diameter of the pipes that are located upstream and downstream of the heat exchanger was increased from 8 to 10 mm. Finally, cavitation conditions were also modelled and avoided in all critical points of the LN_2_ circuit. To validate these modifications, the vibrations of the monochromator with circulating LN_2_ flow were measured using a laser interferometer (Renishaw ML-10 Gold Edition) in the range of frequencies of the LN_2_ flow cryopump of 20–70 Hz, in 1 Hz steps (Fig. 2 ▶). The vibrational modes of the monochromator crystal appeared to be totally decoupled from the cryopump, indicating that the monochromator is vibrationally stable. Other metrology tests show that the first resonance of the monochromator mechanics is well above 150 Hz, which is much higher than the frame rate of the main detector of the beamline (12 Hz).

**Figure 3:**
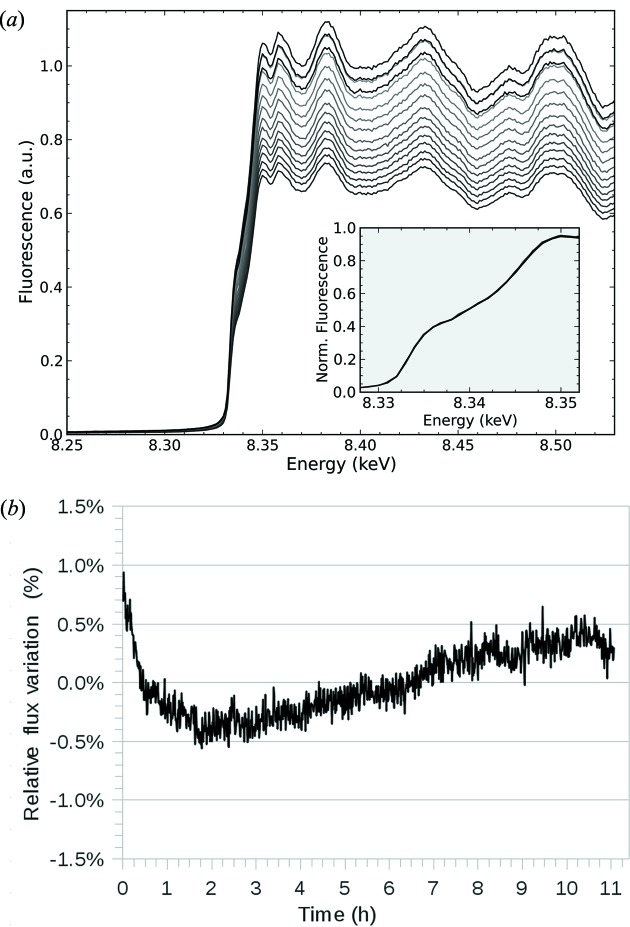
Energy reproducibility and stability of the monochromator. (*a*) Ni *K*-edge (8.333 keV) scans in 1 eV steps collected every 45 min for 11 h, with one electron beam injection in between (darker curves were taken later in time). The reduction in fluorescence signal is due to the decay of the beam current in the storage ring. The dispersion of the first inflection point is 0.1 eV peak-to-valley. The inset shows a zoomed view of the absorption edge, normalized by the storage ring current. (*b*) X-ray fluorescence signal from a Ni foil, normalized by the storage ring current, with the monochromator energy fixed at the first inflection point of the Ni *K*-edge. Fluorescence was measured every second over an 11 h period (every point in the plot is the average of ten consecutive measures). The initial flux variation is explained by the increase of the thermal load when the front-end was open just at the start of the measure.

The energy stability was also tested with X-rays. Reproducibility of the monochromator Bragg axis (with the position loop open) was tested by measuring a series of *K*-edge fluorescence scans from a Ni foil overnight with a four-diode BPM (Fig. 3*a* ▶). We observed that the reproducibility of the scans was excellent, with a dispersion of the energy of the first inflection point of 0.1 eV peak-to-valley. This has to be compared with the Darwin width of the Si (111) crystal at the same energy (∼1.6 eV). The static energy stability of the monochromator was also checked by measuring the fluorescence emitted by the Ni foil with the monochromator set at the *K*-edge inflection point energy (8.333 keV) (Fig. 3*b* ▶). We chose this energy because the current of the diode, which is proportional to the photon flux, shows a maximum variation upon the energy at the inflection point of the edge. These measurements showed that the overnight variation of the fluorescence signal, corrected by the storage ring current, is less than 1.5% peak-to-valley. This percentage corresponds to a maximum energy variation of 0.1 eV, as calculated from the slope of the fluorescence scan at the inflection point, which is the conversion factor between the relative flux and the energy variations. This value is much smaller than the natural Darwin width of the reflection used to monochrome the beam, thus the monochromator is effectively stable in working conditions. Furthermore, stability might be further improved by closing the position loop of the Bragg axis using the encoder.

The energy resolution of the monochromator was measured using a classical pitch scan of the second crystal and deconvoluting the result by its rocking-curve width. The value obtained was Δ*E*/*E* ≃ 1.6 × 10^−4^, for the 5–22 keV energy range. This is very close to the theoretical value of ∼1.5 × 10^−4^ when contributions from the vertical divergence of the undulator and the Darwin width of the first crystal are included.

## 4. Mirror system   

The focusing system consists of a vertical focusing mirror (VFM) and a horizontal focusing mirror (HFM) that are placed orthogonally in a Kirkpatrick–Baez (KB) configuration (Kirkpatrick & Baez, 1948 ▶) in two independent positioning supports manufactured by IRELEC (Saint-Martin-d’Hères, France). The optical surfaces, made by InSync Optics (Albuquerque, NM, USA), are made of silicon and have three stripes (bare Si, Rh coating and Ir coating). The proper selection of the stripes provides a good suppression of the third-harmonic from the monochromator. The optical surfaces are planar, with a length of 300 mm and 600 mm for the VFM and HFM, respectively. The mirrors are elliptically bent in the meridional direction by two independent stepper motors.

**Figure 4:**
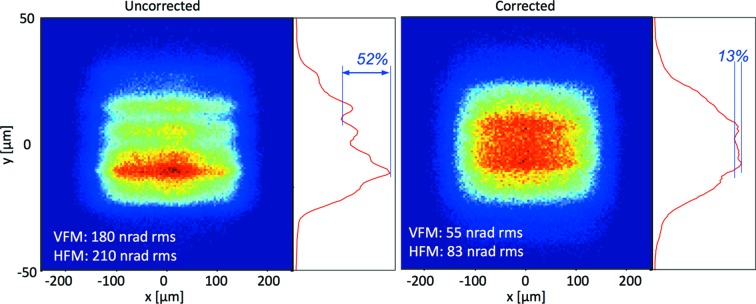
Simulation using ray-tracing program *ART* (Nicolás *et al.*, 2013 ▶) of the beam spot at the sample position when focused at 600 mm downstream. (Left) The beam spot using the original slope error provided by the manufacturer. (Right) The beam spot using the corrected slope error.

The mirror surfaces satisfied the technical specifications when delivered by the manufacturer, with a measured micro-roughness of 2.2 Å for both mirrors, and an RMS slope error of 0.18 µrad and 0.21 µrad at the central Rh-coated stripe, for the VFM and the HFM, respectively. Nevertheless, simulations using the *ART* ray-tracing package (Nicolás *et al.*, 2013 ▶) showed that, while the focal spot remained Gaussian, the defocused beam spot at the sample displayed pronounced striations in the vertical direction when the beam was enlarged fivefold with respect to the focal spot (Fig. 4 ▶). The striations reduced the beam intensity by less than half with respect to the theoretical Gaussian profile. These striations in the beam spot at the sample position were due to the long-period slope errors of the mirror surfaces, which produced secondary foci, together with the small emittance of the photon source (Moreno *et al.*, 2005 ▶). As we can see in Fig. 4 ▶, in order to be able to operate the beamline in defocused mode, these striations of the defocused beam at the sample had to be reduced.

To minimize these striations in the beam, we have developed a new method that corrects large-sized mirrors by using mechanical spring actuators (Nicolas *et al.*, 2013 ▶). The method is based on the classic elastic beam theory and a high-accuracy profile metrology. The classic elastic beam theory provides the analytic relationships between the deformation of the mirror and the set of forces that are applied to it (Goodwine, 2011 ▶). This theory predicts a linear dependence between the surface deformation and each one of the correcting forces, which allows us to decouple the subtle correction of the surface slope errors from the correction of the gravity sag, and these two from the adjustment of the focusing ellipse parameters. The profile metrology measurements were performed at the ALBA-NOM instrument at ALBA, which is equipped with a high-accuracy slope-measuring instrument that is based on the Bessy-NOM concept (Siewert *et al.*, 2004 ▶). The ALBA-NOM can provide measurements that are accurate in the range of a few tens of nanoradians, and well below the nanometer (Nicolas & Martínez, 2013 ▶).

**Figure 5:**
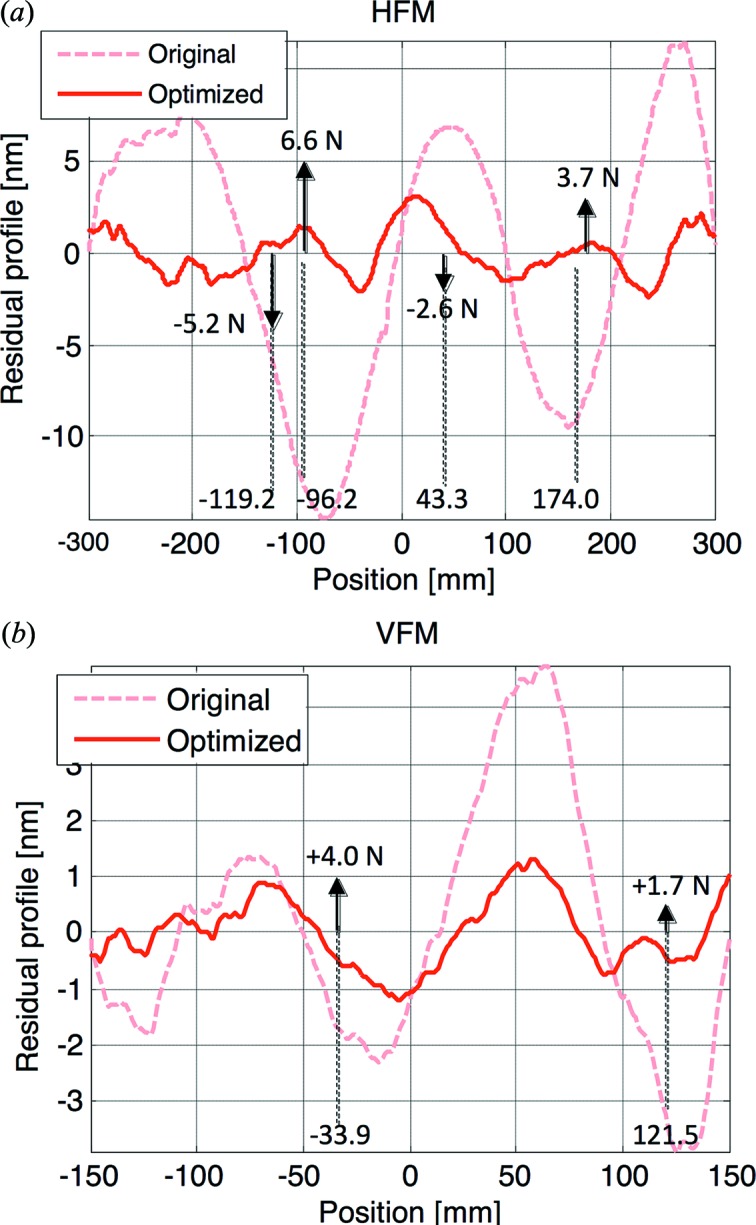
Metrology results of the optimization of the HFM (*a*) and VFM (*b*) of XALOC. The dashed line corresponds to the original height error with respect to the best-fit sphere, while the solid thick line corresponds to the residual profile after optimization. The magnitude of the correcting forces (in N) and position (in mm from the centre of the mirror) are given in the annotations.

For each mirror, we estimated the resulting slope error as a function of the number of correcting forces. The model showed that the RMS slope error was not significantly improved when installing more than two actuators for the VFM, and four actuators for the HFM. Using this number of actuators, our models predicted an improvement of the RMS slope errors from 180 nrad to 55 nrad for the VFM, and from 210 nrad to 83 mrad for the HFM (Fig. 5 ▶). The spring actuators, already present in the mirror bender systems and originally thought only as gravity sag correctors, were adjusted in position and force according to the result of the modelization to correct for the long-period slope errors.

**Figure 6:**
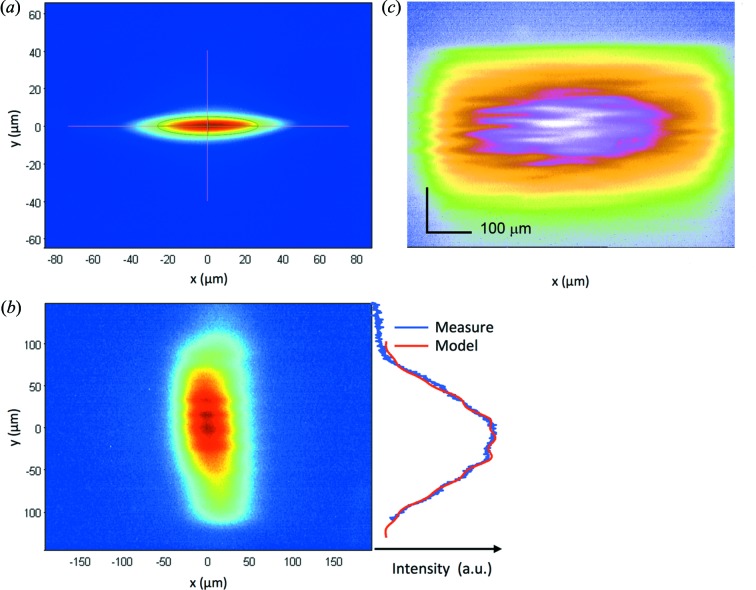
Beam at the sample position using a 20 µm-thick Ce:YAG fluorescence screen. (*a*) Focused beam. (*b*) Vertically defocused beam showing weak stripes. The vertical beam profile is compared with a ray-tracing simulation (including the resolution of the imaging system). (*c*) Beam defocused in the horizontal and the vertical dimensions to 610 µm × 305 µm FWHM (H × V).

**Figure 7:**
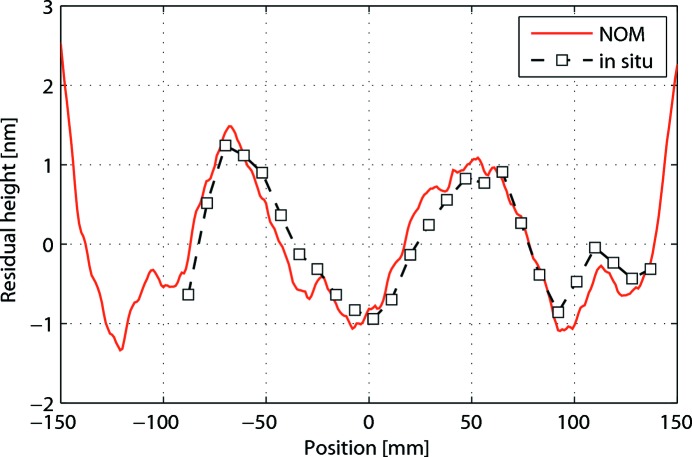
Residual profiles of the VFM in bent working conditions as measured by the ALBA-NOM (continuous line), and as measured *in situ* using the pencil-beam technique using X-ray beam (dashed line).

The effect of the corrected spring actuators was modelled in the final working conditions using the *ART* ray-tracing package and compared with the spot produced by the X-ray beam on a 20 µm-thick Ce:YAG fluorescent screen placed at the sample position (Fig. 6 ▶). The ray-tracing model fully agreed with the measured defocused spot size, showing a Gaussian-shaped vertical profile with minimum striations. The vertical profile striations are the most severe and critical ones for three reasons: (i) the smaller emittance of the source when compared with the horizontal emittance, (ii) the higher defocusing factor required, and (iii) the unhomogeneities along the vertical direction, which is perpendicular to the horizontal oscillation axis, will affect different parts of the crystal at different angles. In order to assess the vertical profile of the slope errors, we checked the VFM by using the pencil-beam method, which provides a direct measurement of the long-period slope errors of the mirror under *in situ* working conditions (Hignette *et al.*, 1997 ▶). The method was carried out by measuring the vertical displacement of the focal spot at the sample position when performing a vertical scan with a set of slits upstream of the mirror at a narrow gap of 10 µm. The calculated slope-error profile using the pencil-beam method fully matched the metrology measurements obtained with the ALBA-NOM two years earlier (Fig. 7 ▶).

As a result of the improvements in the slope errors of the mirrors, the system can defocus the beam vertically by a factor of ten while showing striations that only amount to 10% of the theoretical value. The beam can be used in a very wide range of vertical dimensions at full photon flux. Nevertheless, adjustment of the beam size by focusing or defocusing can only be used in a routine way if the benders modify the beam size without moving the position of the centroid. In XALOC, the position of the centroid deviates by less than 10 µm when defocusing the beam to a vertical FWHM size of 100 µm. This deviation is monitored with a Ce:YAG fluorescent screen at the sample position, and corrected adjusting the diffractometer table.

## 5. Beam characteristics at sample position   

**Figure 8:**
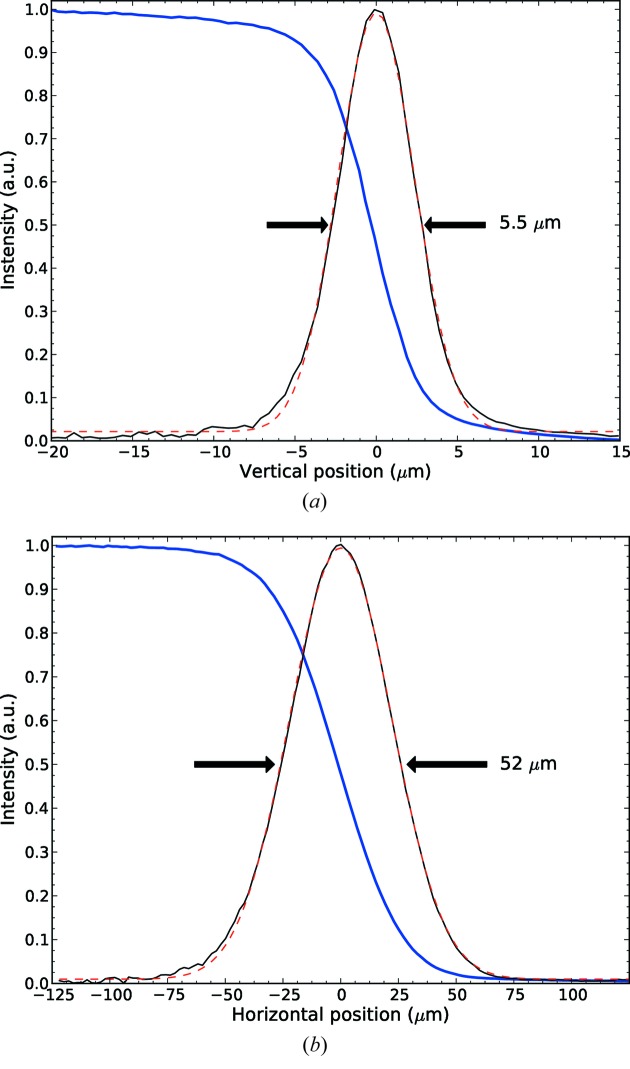
Focused beam size at the sample position in the vertical dimension. The thick and thin solid lines show the raw data and the derivative giving the beam profile, respectively. The dashed line shows the fitting Gaussian.

The X-ray beam is conditioned by the beamline optics described above and propagates to the sample position with excellent characteristics for MX experiments as proven by measures on beam size, flux and stability. The beam size at the sample position (Fig. 8 ▶) was measured using the knife-edge technique by scanning a polished tungsten blade, placed on the diffractometer head, horizontally or vertically through the uncut focused X-ray beam. The derivative of the intensity measured in a Si-PIN diode gave an integrated Gaussian profile in the horizontal and vertical directions with a size of 52 µm × 5.5 µm FWHM. These dimensions match the theoretical beam size expected at the sample position in the absence of significant mirror slope errors, that is, the photon source size divided by the demagnification factor of the corresponding focusing mirror. In this case, the calculated divergence of the focused beam is 600 µrad × 90 µrad (H × V). As shown above, the beam can be easily defocused while preserving the beam centre position and without severe profile striations. As an example, in Fig. 6(*b*) ▶ the beam is defocused vertically to 150 µm with a divergence of 65 µrad and horizontally to 150 µm with a divergence of 570 µrad (all values are FWHM).

**Figure 9:**
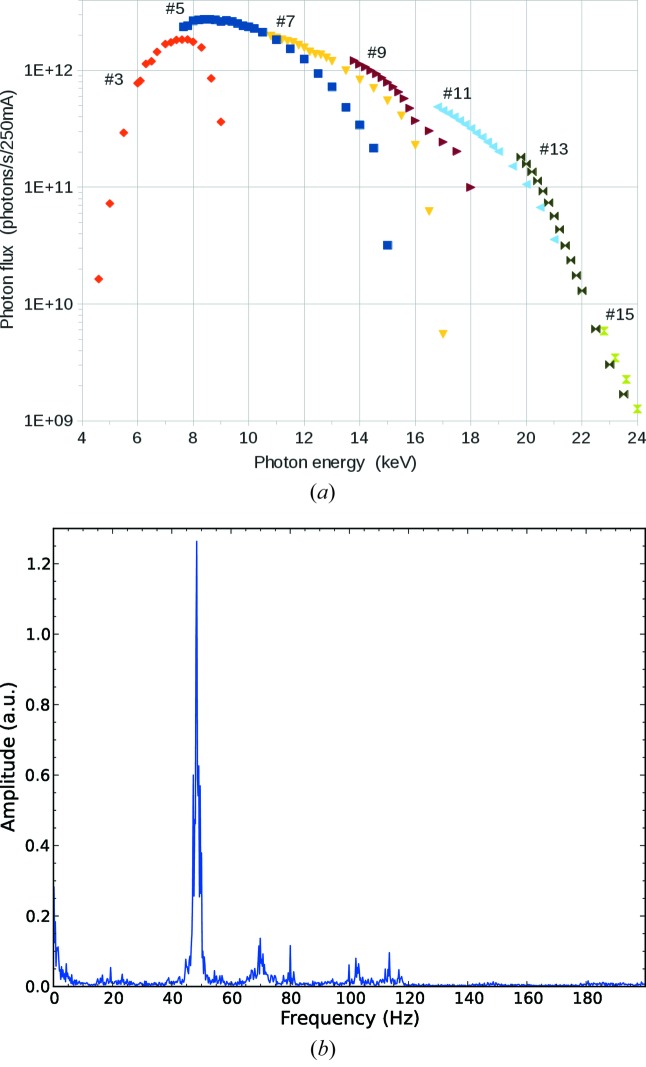
Characteristics of the beam at the sample position. (*a*) Measured flux at the sample position normalized at a current of 250 mA in the storage ring. The numbers represent the harmonic of the undulator. (*b*) Beam vibrational spectrum measured by a Si-PIN diode read at 1 kHz. Note that the maximum frame rate of the detector is 12 Hz.

The flux was measured at the sample position using a calibrated Si-PIN diode AXUV36 (Opto Diode, Newbury Park, CA, USA; formerly IRD) and was found to be above 10^12^ photons s^−1^ when normalized to a current of 250 mA in the storage ring, for a 5–22 keV energy range (Fig. 9*a* ▶).

The stability of the beam at the sample position was assessed by measuring the current of a Si-PIN diode, at a sampling frequency of 1 kHz, that was produced by a beam cut to a size of 1 FWHM using the set of slits closest to the sample (Fig. 9*b* ▶). A first resonance peak was found at 50 Hz, which is likely to be due to electronic noise. Other peaks are found at 60–70 Hz, which were already observed in the vibrational tests of the VFM. The frequencies of the flux oscillations are much higher than the usual frame rate used at the beamline (1–10 Hz). The apparent variation of the flux, including the dominant peak at 50 Hz, amounts to less than 0.5% of the flux. These limited spatial oscillations of the beam, with high frequency and small amplitude, are unlikely to affect significantly the data quality of the diffraction experiments.

**Figure 10:**
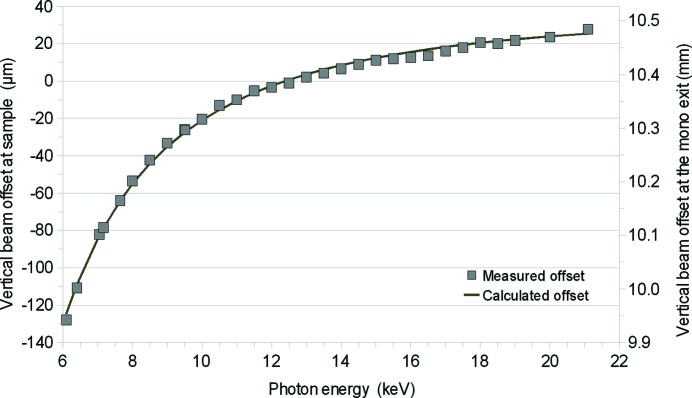
Vertical position of the beam depending on the photon energy at the sample position (left axis) and at the mono exit (right axis). The modelled curve with a gap between crystals of 5.253 mm (line) is in very good agreement with the measured position of the beam as measured with a fluorescing screen at the sample position (squares).

The channel-cut monochromators are known to change the position of the exit beam in the dispersive direction (*i.e.* vertical in XALOC) depending on the photon energy. In the case of our monochromator, the vertical excursion of the beam at the sample position is only ∼150 µm in the 6–22 keV energy range as measured using a fluorescent screen. This excursion is actually smaller for the most common energy changes; for example, between the Pt *K*-edge (11.564 keV) and the Se *K*-edge (12.658 keV) the excursion is 30 µm. The vertical excursion of the beam at sample position *h* is well explained by the expression
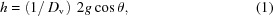
where *D*_v_ is the demagnification of the source by the VFM (which equals to 3.46) and the factor (2*g* cosθ) is the well known expression for the beam excursion after diffraction on two parallel crystals. In this expression, *g* is the distance between the surfaces of the monochromator (specified to be 6 mm) and θ is the Bragg angle of the (111) reflection. The vertical excursion of the beam as measured at the sample position is reproduced by the above expression if the gap between crystals is set to 5.25 mm (Fig. 10 ▶). The discrepancy between the theoretical value of the gap and the measured one can be attributed to the manufacturing tolerance and to the misalignment of the axis Bragg angle with respect to the surface plane of the first crystal. The dependence of the vertical position of the beam with respect to the photon energy is reproducible and is corrected automatically using a look-up table to adjust the sample and detector positions at every energy change. The beam shows a typical vertical long-term drift of 0.5–1 µm h^−1^ downwards, which is corrected adjusting the diffractometer table. The drift is totally correlated with the refilling and the current decay of the storage ring, and consequently we expect the drift to be eliminated when the storage ring is operated in top-up mode in the near future.

## 6. End-station   

**Figure 11:**
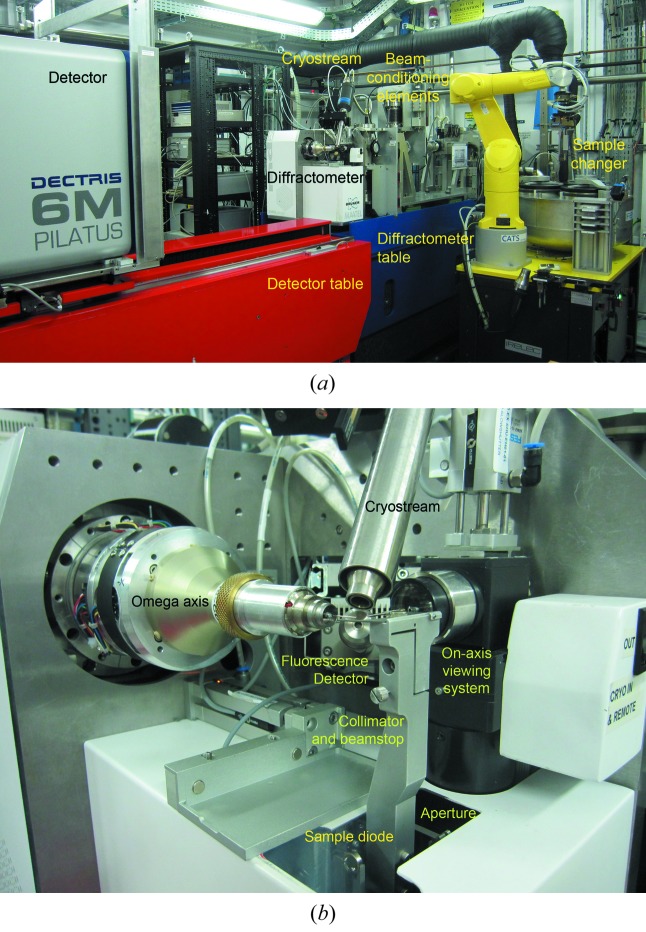
(*a*) General view of the BL13-XALOC end-station. The beam-conditioning elements include, as seen following the beam, an XBPM, 12 in-line attenuators, a fast shutter, a second XBPM, a set of four-blade slits and a second XBPM. (*b*) Close-up of the sample environment of BL13-XALOC showing the mounted mono-axis (ω), the beamstop, the beam-shielding capillary and cleaning aperture, the cryostream, the fluorescence detector, the LN_2_ cover, and the pneumatically actuated shutter. The sample diode and the 30 µm aperture are retracted.

The layout of the end-station (Fig. 11*a* ▶) is based on two high-precision positioning tables that place independently the diffractometer and the detector. The mechanical and vibrational behaviour of the diffractometer table is particularly important for the operation of the beamline because it is necessary to accurately and reliably align the sample with the X-ray beam after changing the photon energy or the optical configuration (*i.e.* going from focused to unfocused mode).

The positioning tables were designed at ALBA and are based on flexures that work in non-relaxed conditions for the pitch and yaw rotations. They are designed using the so-called *skin* concept, where the mechanics is built around a granite support and as close as possible to the axis of the X-ray beam (Colldelram *et al.*, 2010 ▶). The resulting mechanical performance of the diffractometer table is outstanding, reaching a resolution equal or better than 0.5 µm for the translations perpendicular to the beam axis, and better than 0.5 µrad for their respective rotations (pitch and yaw). Repeatabilities in open loop are also in the range of 1 µm for the translations and 1 µrad for the rotations. The movement ranges are large for translations (>60 mm) and short for rotations (15 mrad) due to the elastic limit of the flexure. The design of these tables, which introduces massive links for rotational adjustments instead of friction-based links such as kinematical mounts, results also in an excellent vibrational behaviour (their first resonant mode appears at 43 Hz). Similar values are found for the less-critical detector table.

The beam-conditioning elements and diagnostics on the diffractometer table consist of a set of 12 attenuators in tandem (seven Al foils with thicknesses in the range 7–500 µm, Fe 7 µm, Ni 7.5 µm, Zn 10 µm, Au 5 µm, Zr 25 µm) that adjust the transmission of the X-ray beam, a 2 ms-aperture time shutter FPS400M (fast shutter) from CEDRAT (Meylan Cedex, France), a set of vertical and horizontal slits AT-F7-HV (JJ-X-ray, Lyngby, Denmark), two CVD diamond four-quadrant BPMs XBPM4-S (DECTRIS Ltd, Baden, Switzerland), and a pneumatic shutter manufactured at ALBA. The vacuum chambers and positioning tables for the fast shutter, slits and BPMs were designed at ALBA.

The sample-viewing system consists of an on-axis parallax-free high-resolution video microscope (0.23 µm pixel^−1^ at maximum zoom) and user-controlled front and polarized back lights (transmitted). The single-axis (ω) diffractometer MD2M (Maatel-Bruker, Moirans, France) (close-up view in Fig. 11*b* ▶) shows a repeatability of 0.048 mdeg and a RMS following error of 0.2 mdeg, measured using a laser interferometer, and a sphere-of-confusion (SOC) of around 1 µm, as measured using the on-axis microscope at maximum zoom. The measured SOC of the ω axis is in agreement with the reported value for this diffractometer (Cipriani *et al.*, 2007 ▶). If needed by the user, a mini-kappa mount (MK3) that adds two extra degrees of rotation (ϕ and κ), can be easily mounted on the diffractometer. Although the MK3 is useful to collect complete data at high resolution and to optimize anomalous data collections, it increases the SOC to <5 µm. The sample environment of the MD2M diffractometer is composed of a 700 µm-diameter beamstop, a 2 cm-long beam-shielding capillary (790 µm internal diameter) with a 350 µm-internal-diameter cleaning aperture made of Pt, and a 30 µm Pt beam-defining aperture [that can collimate the beam to 20 µm × 5.5 µm FWHM (H × V) at the sample position]. The crystals are kept at 100 K with an Oxford Cryostream 700 (Oxford Cryosystems, Oxford, UK) held in place by a modified in-house version of its positioning table that is actuated with a stepper motor. Sample centring is performed *via* the on-axis-viewing system widget (a.k.a. OAV). This widget allows us to centre the sample by employing a three-click-centring procedure and to automatically focus the sample. With this widget we are also able to control many important motors of the beamline, namely the ω and the MK3 axes. An ellipse on the crystal video frame shows the zoom-dependent FWHM size of the beam. It is planned to employ the OAV widget to implement helical data collection and sample centring strategies using diffraction. It is also foreseen to install a selection of pinholes that range from 5 µm to 30 µm in diameter to further reduce the horizontal beam size.

A cryogenic automated transfer system robot, or CATS (IRELEC, Saint-Martin-d’Hères, France) (Ohana *et al.*, 2004 ▶), that handles cryogenic samples mounted in standard SPINE pins is also available at the beamline. The robot can read the standard dot matrix barcode printed in the caps for easy sample tracking. The cryosamples are stored in a LN_2_ Dewar that can store 90 SPINE vials/caps. CATS operates reliably with a low failure rate of <1% over 3000 mounted samples, provided that the relative humidity in the hutch is less than 30%. The samples can be mounted in different positions of the diffractometer, coping with the adjustment in position due to the photon energy or the optical configuration. Removing a sample from the goniometer, picking a new frozen sample from the LN_2_ Dewar and transferring it to the goniometer takes about 40 s. The robot and the sample environment are monitored by the user *via* Ethernet webcams and VLC viewers. The robot is also prepared to handle Greiner crystallization plates. However, the *in situ* diffraction experiments are not still routinely available since the set-up requires a beamstop that can be also moved along the beam and in a different configuration than the one that came with the MD2M. The installation of this beamstop, with exchangeable pellets with different diameters, is foreseen in the near future.

The main data collection detector is a photon-counting Pilatus 6M (DECTRIS Ltd, Baden, Switzerland) (Broennimann *et al.*, 2003 ▶) that can be placed at 123.5–1356 mm from the sample and is operated in shutterless mode. The Pilatus 6M detector offers a large sensitive area (431 mm × 448 mm), a fast framing rate (12.5 Hz), a large dynamic range (20 bits), a negligible dark-current noise, a point spread function of 1 pixel, and the possibility of applying an energy threshold to the collected data (Kraft *et al.*, 2009 ▶). A NI6601 digital I/O card (National Instruments, Austin TX, USA) is responsible for the synchronization of the ω-angle rotation, the fast shutter and the detector.

In order to obtain fast and accurate XANES spectra and to select the optimal X-ray energy for wavelength-dependent experiments, the beamline is equipped with a Si-drift fluorescence detector X-Flash 410 (Bruker AXS Microanalysis GmbH, Berlin, Germany) that is positioned using a retractable pneumatic table. A second Si-drift fluorescence detector XR-100SDD (AMPTEK, Bedford, MA, USA) is also available as a backup option. Typically a fluorescence scan takes ∼100 s and the fluorescence data are processed automatically after the scan with *CHOOCH* (Evans & Pettifer, 2001 ▶). Faster on-the-fly scans will be available in the near future.

The beamline can be easily aligned by using a 20 µm-thick Ce:YAG fluorescent screen. The beam alignment involves only the positioning tables of the end-station; the focusing mirrors are not moved when changing the energy, as the beam footprint is included in the mirror acceptance. The alignment procedure (even after extended shutdowns) takes less than 5 min when we go from 7.1 keV to 15 keV and about 10 min from 5 to 22 keV (because it involves changing the stripes of the mirrors to other than the standard Rh coating). It takes on average ∼2–3 min to focus or defocus the beam to any dimensions of the beam from 52 µm × 5.5 µm to 300 µm × 300 µm FWHM (H × V) (see Video 1 of the supporting information for a real-time defocusing–focusing cycle in both dimensions^1^). The maximum size of the beam is limited by the cleaning aperture in the beam-shielding capillary. These operations, that can also be performed by users, will be automated in the near future, thus reducing even further the time required to complete them. Another automated method to align the beam at the sample position will be implemented based on a pinhole aperture and a PIN diode. Alternatively, to unfocus the beam (*i.e.* to remove one or both of the focusing mirrors) takes 45 min and it can only be done by a beamline scientist. This unfocused mode gives us, however, a large beam without striations with very low divergence in the vertical dimension (25 µrad). The size of the unfocused beam is ∼2 mm × 0.7 mm (FWHM) (H × V), although the beam size is in practice limited to 300 µm in either dimension by the diameter of the cleaning aperture. This mode has been tested during commissioning but has not yet been used by the users.

A control hutch near the end-station is equipped with one Linux workstation that is used to operate the beamline and visualize on-line the diffraction data. Two additional Linux workstations and one Windows-based PC are used for diffraction data processing, crystal structure determination and for automated data backup. The diffraction strategy is calculated by *EDNA* (Incardona *et al.*, 2009 ▶) and all the common processing software packages in MX are available to the users. Users’ data are stored at ALBA for six months and can be accessed *via* a VPN-based secure access service.

If required, crystal samples can be prepared and/or mounted in a small sample preparation area that is located right outside of the end-station. The users can also use a larger biology laboratory close to the beamline to prepare their samples, buffers or even perform crystallizations. This larger laboratory is equipped with tabletop centrifuges, microcentrifuges, stirrers, microscopes, crystallization incubators, glassware, cryotools, fridge/freezers, one fume hood, and an equipped large cold room for samples that need to be at 277 K.

## 7. Control system and automation   

**Figure 12:**
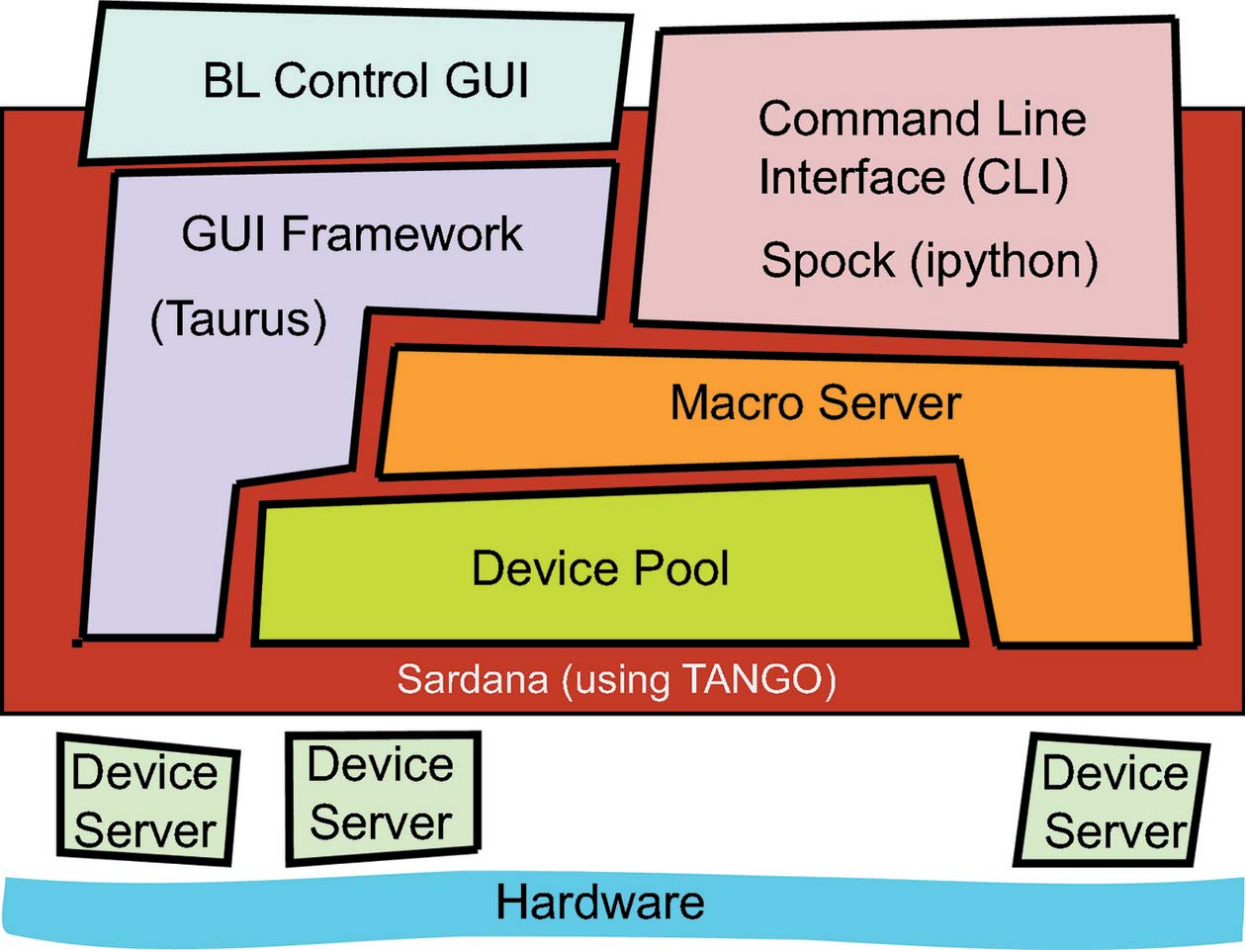
Block diagram describing the architecture of Sardana, the control system of the BL13-XALOC beamline.

The beamline control system is based on Sardana, a new supervision, control and data acquisition (SCADA) package inspired in the TANGO collaboration (Coutinho *et al.*, 2011 ▶). The advantages of Sardana are a powerful Python-based environment for building and executing macros defined in the macro server, a comprehensive and optimized access to the hardware, a standard command-line interface based on iPython called SPOCK, and a generic and customizable graphical user interface (GUI) using the Taurus library (Fig. 12 ▶). A major feature of Sardana is that the controlled elements (hardware, or any other control entity) can be defined in a device pool through controllers that interface the diversity of hardware (*e.g.* different motor manufacturers) with standard control methods (*e.g.* motor positions or limits are read in a unique way). The control architecture above the pool is consequently not affected by changes in hardware. Currently, standard MX experiments are performed through independent widgets which control the beamline instrumentation (sample-viewing system, automatic sample changer, fluorescence scans, *etc.*) and share the same device pool and macro server. In the near future the widgets will be grouped together in order to implement higher-level beamline automatizations like batch or inverse-beam data collections. Remote data collection is going to be implemented in the forthcoming year.

## 8. First results   

During the commissioning, the beamline was tested using a variety of protein crystals and techniques. The selection of results shown in Table 2 ▶ demonstrates that the beamline is able to deal with a large variety of data-collection scenarios. These scenarios range from standard X-ray diffraction experiments that tend to be automated to experiments that require more human intervention like dealing with microcrystals or large unit cells. After this successful commissioning period of only two months between the first test crystal and the start of user operation, BL13-XALOC received its first users on 18 July 2012 performing MX wavelength-dependent (MAD/SAD) and robot-assisted experiments from the first day. Since then, we have been able to exploit the anomalous signals from a variety of *K* (S, Cd, Mn, I, Fe, Ni, Cd, Cu, Zn, Se and Br) and *L*_III_ (Ho, Os, Sm, Gd, Ir, Pt, Au and Hg) absorption edges. From the start of user operation until March 2014, 60 research groups have tested 8363 crystals and collected 3281 datasets (defining one dataset as having 90 or more image frames), which has allowed the users to solve a large variety of structures that include integral membrane proteins or large-unit-cell crystals. As a result, the scientific production has started in earnest with the first structures being deposited in the Protein Data Bank and the first papers being published in peer-reviewed journals (Bacarizo & Camara-Artigas, 2013 ▶; Gallego *et al.*, 2013 ▶; Gallego del Sol & Marina, 2013 ▶; Otero *et al.*, 2013 ▶).

## Supplementary Material

Video 1. Short movie showing the beam spot at the sample position when changing the bending radius of the vertical focusing mirror from focal position to defocused position (5.5 to 100 micrometre FWHM). No corrections or realignments are made during the scan.. DOI: 10.1107/S160057751400825X/rv5017sup1.avi

A diffraction frame from crystals of Human Rhinovirus 2 (N. Verdaguer) taken with the Pilatus 6M detector. The data collection parameters are listed in Table 2.. DOI: 10.1107/S160057751400825X/rv5017sup2.pdf

## Figures and Tables

**Table 1:** Characteristics of the photon source and optical elements

Undulator maximum magnetic field	0.806 T
Undulator period	21.6 mm
Number of magnetic periods	92
Photon source size	310 µm × 18 µm FWHM (H × V)
Photon source divergence	112 µrad × 25 µrad FWHM (H × V)
Undulator power at 250 mA in storage ring	1.8 kW
Monochromator type	Channel-cut double-crystal silicon, using [111] planes
Energy range (wavelength range)	5–22 keV (2.3–0.55 Å)
Vertical focusing mirror (VFM)	Elliptically bent silicon mirror, with three stripes (Si, Rh, Ir); optical length 300 mm, incident angle 4 mrad; 55 nrad RMS slope error (corrected slope)
Horizontal focusing mirror (HFM)	Elliptically bent silicon mirror, with three stripes (Si, Rh, Ir); optical length 600 mm, incident angle 4 mrad; 83 nrad RMS slope error (corrected slope)

**Table 2:** A selection of the first diffraction data collections taken during beamline commissioning *t* stands for exposure time and Δω for oscillation angle per image. The storage ring current was between 60 and 90 mA. Values in parentheses are data for the lowest-resolution shell. ISa refers to *I*/σ(*I*)^asymptotic^, as defined by Diederichs (2010 ▶).

Sample	Space group, cell parameters (Å, °)	Total oscillation (°)	Resolution range (Å)	*R*_sym_ (%)	Comments and references
Hen egg-white lysozyme	*P*4_3_2_1_2, *a* = *b* = 79, *c* = 37	90	1.26 Å (3.76 Å)	3.8% (2.0%)	ISa = 27. First high-quality data, λ = 0.979 Å, *t* = 1 s, Δω = 1°
*Gallus gallus* SH3 mut of c-Src tyr kinase	*P*3_1_2_1_, *a* = *b* = 31.5, *c* = 106.8	90	0.93 Å (2.34 Å)	3.9% (3.6%)	ISa = 21. Atomic-resolution data, λ = 0.827 Å, *t* = 2.6 s, Δω = 0.5° (Bacarizo & Camara-Artigas, 2013 ▶)
*D. mobilis* meganuclease I-Dmol	*P*21, *a* = 106.6, *b* = 70.3, *c* = 107.1, β = 119.9	360	2.40 Å (6.0 Å)	6.9% (3.8%)	ISa = 21. First Se-SAD dataset, λ = 0.979 Å, *t* = 1 s, Δω = 1° (crystals from G. Montoya, CNIO, Spain)
Human Rhinovirus 2 with inhibitor	*C*2, *a* = 464, *b* = 374, *c* = 461, β = 98.8	187	4 Å (11.7 Å)	14.4% (5.5%)	ISa = 20. Large unit cell. Sample–detector distance 752 mm. λ = 0.979 Å, *t* = 1 s, Δω = 0.5°† (crystals from N. Verdaguer, IBMB, Spain)

† See Fig. S1, one diffraction image, in the supporting information.

## Footnotes

1 Supporting information for this paper is available from the IUCr electronic archives (Reference: RV5017).
