# Supplementary material for: Developments in optics and performance at BL13-XALOC, the macromolecular crystallography beamline at the Alba Synchrotron
Source: J Synchrotron Radiat. 2014 May 20;21(Pt 4):679–89. doi: 10.1107/S160057751400825X (PMC4073956; doi:10.1107/S160057751400825X)
Supplement: Supplementary file 2 [file s-21-00679-sup2.pdf]

Supporting information for JSR paper RV5017

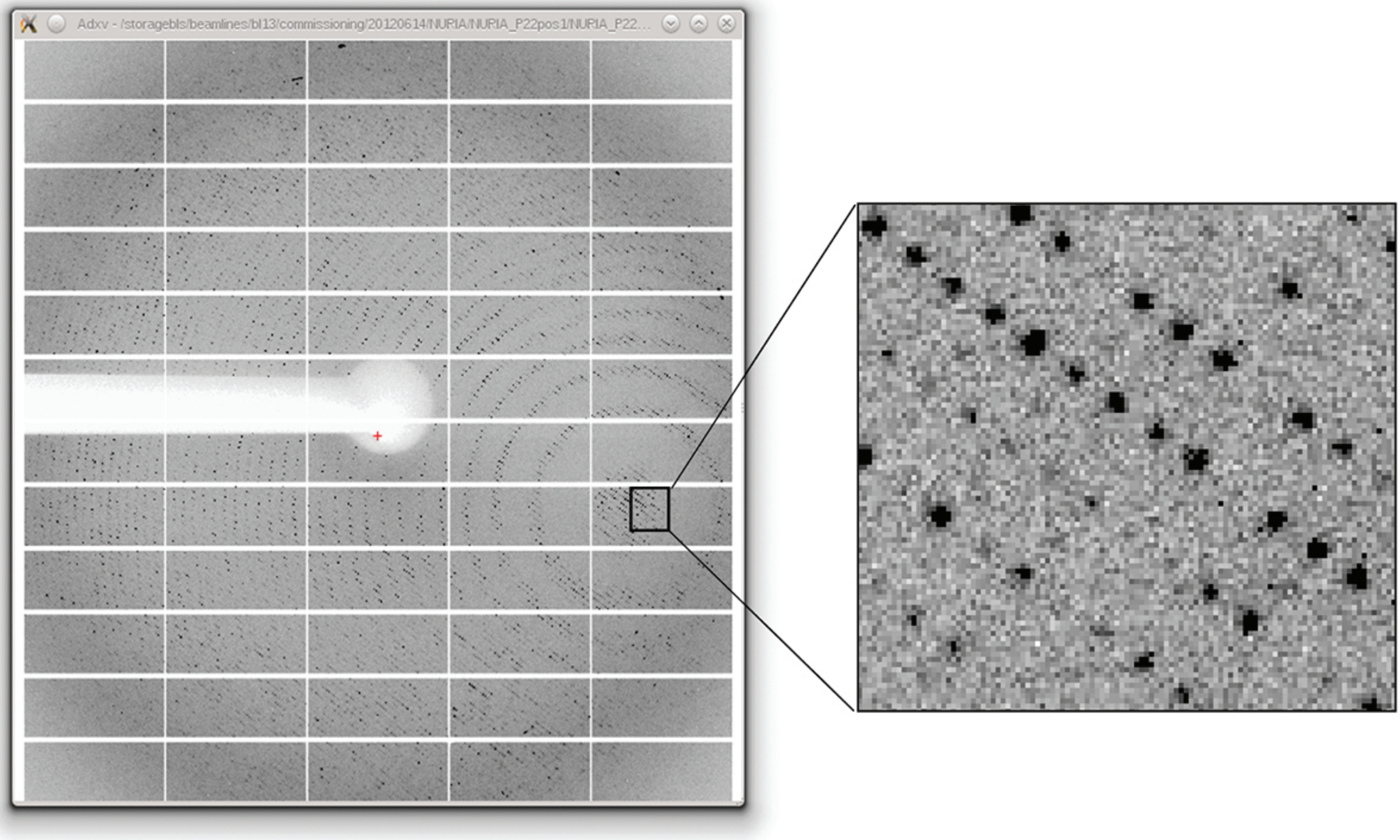

Fig. S1 A diffraction frame from crystals of Human Rhinovirus 2 (N. Verdaguer) taken with the Pilatus 6M detector. The data collection parameters are listed in Table 2 of the paper.
